# Nelfinavir and other protease inhibitors in cancer: mechanisms involved in anticancer activity

**DOI:** 10.12688/f1000research.5827.2

**Published:** 2015-03-05

**Authors:** Tomas Koltai

**Affiliations:** 1 Centro de Diagnostico y Tratamiento de la Obra Social del Personal de la Alimentación, Talar de Pacheco, Buenos Aires, 1618, Argentina

**Keywords:** Nelfinavir, protease inhibitor, cancer, endoplasmic reticulum stress, unfolded protein response, Akt

## Abstract

**Objective:** To review the mechanisms of anti-cancer activity of nelfinavir and other protease inhibitors (PIs) based on evidences reported in the published literature.

**Methods:** We extensively reviewed the literature concerning nelfinavir (NFV) as an off target anti-cancer drug and other PIs. A classification of PIs based on anti-cancer mode of action was proposed. Controversies regarding nelfinavir mode of action were also addressed.

**Conclusions:** The two main mechanisms involved in anti-cancer activity are endoplasmic reticulum stress-unfolded protein response pathway and Akt inhibition. However there are many other effects, partially dependent and independent of those mentioned, that may be useful in cancer treatment, including MMP-9 and MMP-2 inhibition, down-regulation of CDK-2, VEGF, bFGF, NF-kB, STAT-3, HIF-1 alfa, IGF, EGFR, survivin, BCRP, androgen receptor, proteasome, fatty acid synthase (FAS), decrease in cellular ATP concentration and upregulation of TRAIL receptor DR5, Bax, increased radiosensitivity, and autophagy. The end result of all these effects is slower growth, decreased angiogenesis, decreased invasion and increased apoptosis, which means reduced proliferation and increased cancer cells death.

PIs may be classified according to their anticancer activity at clinically achievable doses, in AKT inhibitors, ER stressors and Akt inhibitors/ER stressors.

Beyond the phase I trials that have been recently completed, adequately powered and well-designed clinical trials are needed in the various cancer type settings, and specific trials where NFV is tested in association with other known anti-cancer pharmaceuticals should be sought, in order to find an appropriate place for NFV in cancer treatment.

The analysis of controversies on the molecular mechanisms of NFV hints to the possibility that NFV works in a different way in tumor cells and in hepatocytes and adipocytes.

## Abbreviations

NFV: Nelfinavir

PI: HIV Protease Inhibitors

ERS: Endoplasmic reticulum stress

UPR: Unfolded protein response

BCRP: Breast cancer resistance protein

FAS: Fatty Acid Synthase

## Introduction

In March 1997, the United States Food and Drug Administration (FDA) approved Nelvinavir (NFV, brand name Viracept) for HIV treatment in humans ^1^. NFV is a safe, orally available, and potent drug against HIV-1 and HIV-2 ^2^. This protease inhibitor (PI) was developed by the private pharmaceutical sector and was a big success in the treatment of AIDS in association with other anti-retroviral drugs ^3^. The introduction of PIs combined with HIV reverse transcriptase inhibitors started the era of HAART (highly active anti-retroviral treatment) and is nowadays the standard of care in HIV-AIDS ^4^.

PIs inhibit HIV-1 and HIV-2 proteases (which are aspartate proteases), impeding virus replication and release of infecting viral particles from diseased cells. The mechanism of action of protease inhibitors involves competitive binding to the enzyme ^5^.

NFV is being progressively displaced from HIV therapeutics by second generation HIV PIs, but has shown interesting off target actions in cancer.

The possible use of anti-HIV drugs against cancer is not new: in the 1990s AZT (zidovudine or azidothymidine) was proposed as anti-neoplastic drug, but clinical trials did not confirm the preliminary good results obtained *in vitro* ^6^.

That HIV PIs target other molecules besides the HIV protease is quite evident if we examine adverse effects like insulin resistance and lipodystrophy. These and other evidences such as inhibition of tumor cell production of cytokines, anti-angiogenesis, induction of apoptosis and others, suggest off targets effects for PIs, and hints to the concept of a new class of drugs against cancer with multiple anti-cancer effects ^6^.

NFV, the most important anti-cancer drug of the PI family, if repurposed for cancer treatment, would have an important advantage: it has been used for more than 15 years in HIV treatment and its safety, pharmacokinetics, and adverse events are well known. Serious adverse events are not common with the exception of diarrhea when used at high doses.

Research on NFV as a potentially useful drug for cancer treatment ^6^ started in 2009.

In this article, we thoroughly review the literature published in this matter and analyze mainly the anti-cancer mechanisms of action of NFV.

Certain controversies regarding NFV activity in lipid metabolism will be considered in depth.

## Evidences of nelfinavir anti-cancer activity

A partial response of Kaposi’s sarcoma patients to PIs was published in 1998 ^7^ and good results with regression (six complete responses out of 10 patients) ^8^. In 1999 Niehues *et al.* ^9^ published complete regression of Kaposi’s sarcoma in a child treated with highly active anti-retroviral therapy (HAART). Sgadari *et al.* (2003) described also the inhibition of Kaposi’s sarcoma with protease inhibitors and they also mention that these drugs can antagonize vital properties of tumor cells like growth, invasion, tissue remodelling, angiogenesis and survival. They consider these effects to be a consequence of inhibition of invasion, matrix metalloprotease, proteasome and NF-κB signaling ^10^. The possible mechanisms of PIs off target activity on tumor cells were described by pioneering work of Schmidtke *et al.* in 1999 ^11^: they observed that ritonavir was a modulator of proteasomal activity, allowed normal proliferation when used at low concentrations, but affected protein degradation when present at higher concentrations, and cell cycle was arrested.

Ikezoe *et al.* ^12^ described that protease inhibitors increased cellular growth inhibition of all transretinoic acid (ATRA) on cell cultures of myelocitytic leukaemia lines. Protease inhibitors also increased differentiation of acute myeloid leukemia cell lines.

In 2004, Ikezoe ^13^ described the mechanisms involved in anti-cancer activity of protease inhibitors in myeloma cells.

The mechanisms involved in PIs anti-cancer activity are summarized in chronological order on Table 1.

**Table 1.** Mechanisms of action of Nelvinavir and other PIs in cancer.

Author	Study performed in	Results
Andre, 1998 ^14^	Mice infected with lymphocytic choriomeningitis virus receiving ritonavir.	Ritonavir inhibits chymotrypsin-like activity of the 20S proteasome. Nelfinavir does not inhibit chymotrypsin like activity.
Gaedicke, 2002 ^15^	Thymoma cells growing in syngeneic mouse	Ritonavir produces growth inhibition of tumors, apoptosis and affects proteosomal proteolysis. Non-transformed cell lines were relatively resistant to this activity. Accumulation of p21 (due to inhibition of proteolytic degradation).
Ikezoe, 2004 ^13^	Human Multiple Myeloma cells	Growth arrest, apoptosis, blocked IL6 stimulated phosphorylation of STAT 3 and ERK ½. Decreased VEGF production.
Sgadari, 2002 ^16^	Kaposi sarcoma cell lesions in nude mice	Anti-angiogenesis. Decrease of VEGF, bFGF. Decrease MMP2 activation.
Pajonk, 2002 ^17^	PC-3 and DU-145 prostate cancer, U373 glioblastoma, and K562 and Jurkat leukemia cells	Saquinavir inhibited activation of NF-κB. Inhibited 20s and 26s proteasome activity. Sensitized the surviving cells to ionizing radiation. In a previous paper ^18^, this same group showed that proteasome is a direct target of radiation. This explains the synergism between proteasome inhibitors and ionizing radiation.
Olson, 2002 ^19^	Cell culture of UMCC-1/VP cells which over-express MRP-1	Ritonavir inhibits the functional activity of the multidrug resistance related- protein 1 (MRP-1). This characteristic was not shared by other PIs.
Zhou, 2004 ^20^	Insulinoma cells	Nelfinavir decreased insulin stimulated phosphorylation of IRS-2 and Akt- Thr(308) in a dose-dependent manner. For 10 micromol/L of nelfinavir, the decrease in Akt phosphorylation was 55%.
Gupta, 2004 ^21^	Human embryonic kidney cells	HIV PIs are breast cancer resistance protein inhibitors. This applies to ritonavir, saquinavir, and nelfinavir. Indinavir and amprenavir showed no inhibition on BCRP.
Piccinini, 2005 ^22^	HL60 cells incubated with and without drug.	Saquinavir and nelfinavir inhibited proteasome activity at therapeutic dosages. Retroviral medication had no effect on proteasome.
Gupta, 2005 ^23^	Tumor cell culture and xenografts.	Amprenavir, nelfinavir, and saquinavir inhibited Akt phosphorylation and exerted synergistic effects with radiotherapy.
Yang, 2005 ^24^	Prostate Cancer Cells: LNCaP, DU145, PC3 and LNCaP xenografts in nude mice	NFV induces growth arrest and apoptosis of prostate cancer cells and blockade of androgen receptor, STAT3 and AKT. It also inhibits proliferation of LNCaP xenografts.
Yang, 2006 ^25^	NSCLC cell culture and xenografts in nude mice.	NFV induces growth arrest, reduces Akt signalling, apoptosis and docetaxel sensitisation. It is responsible for up-regulation of p21, p27 and p53, down- regulation of Bcl-2 and MMP-2. NFV slowed proliferation and induced apoptosis in tumour xenografts mice without adverse systemic effects. Of the 3 PIs tested (saquinavir, ritonavir and NFV) NFV exerted the strongest inhibition on proliferation.
Chow, 2006 ^26^	Liposarcoma and non liposarcoma cell lines	NFV induces apoptosis of liposarcoma cell through up-regulation of SREBP-1. Authors consider that NFV is a new class of anti-liposarcoma agent.
Pore, 2006 ^27^	Glioblastoma cells	NFV decreased VEGF expression and secretion under normoxia. NFV decreases VEGF through the PI3K/Akt pathway. NFV also decreased the hypoxic induction of VEGF and the hypoxic induction of HIF-1alpha. NFV’s effect was a decreased angiogenesis
Pore, 2006 ^28^	*In vivo* Matrigel plug assay	NFV decreases VEGF expression through the transcription factor Sp1, which regulates VEGF promoter. It down-regulates HIF-1 alfa by decreasing translation.
Hampson, 2006 ^29^	HPV transformed cervix carcinoma cells	Protease inhibitors inhibit S 26 proteasome blocking p53 degradation.
Ben-Romano, 2006 ^30^	Cell culture of 3T3-L1 adipocytes	NFV induces oxidative stress that may lead to apoptosis (in adipocytes).
Gupta, 2007 ^31^	Meningioma cells	Combination therapy with imatinib and NFV potentiated anti-proliferative activity of imatinib due to decrease in survivin and increase of Bax.
Jiang, 2007 ^32^	Melanoma cells	NFV produces cell cycle arrest and apoptosis through inhibition of CDK2.
Jiang and Pore, 2007 ^33^	Glioblastoma cells	NFV decreased Akt expression and enhanced radiosensitization in PTEN deficient glioblastoma cells.
Gills, 2007 ^34^	NSCLC xenografts and breast cancer resistant cell lines	NFV induced caspase dependent apoptosis and also caspase independent apoptosis via ER stress and autophagy.
Cuneo, 2007 ^35^	HUVEC and tumor vascular endothelium	NFV enhance the effects of irradiation on endothelial cells.
Pyrko, 2007 ^36^	Glioblastoma cell lines	Endoplasmic reticulum stress (ERS) response, as shown by increased expression of ERS markers, GRP78 and CHOP, and activation of ERS- associated caspase-4. Proteasome inhibition.
De Barros, 2007 ^37^	Human subcutaneous abdominal white adipose tissue	PIs inhibited proteasome and differentiation of human preadipocytes in culture, reducing expression and production of matrix metalloproteinase 9 (MMP-9).
Gills, 2008 ^38^	60 different cancer lines	NFV causes apoptosis and non-apoptotic death, ERS and autophagy. Blocks growth factor receptor activation and decreases growth factor-induced and endogenous Akt signaling. *In vivo*, NFV inhibits tumor growth.
Plastaras, 2008 ^39^	Leucocytes of HIV patients receiving PI (peripheral blood biomarker assay)	Decreased Akt activation at clinically achievable doses. Increases sensibility of cancer cells to radiotherapy. PIs do not increase toxicity in patients receiving radiotherapy.
Brüning, 2008 ^40^	Ovarian cancer cells	NFV upregulates TRAIL receptor DR5 which is an apoptosis inducing receptor.
Giri, 2009 ^41^	MDCKII wild-type and Bcrp1- transfected cell lines	NFV may act as a breast cancer resistance protein (BCRP) inhibitor with certain substrates.
Brüning, 2009 ^42^	Ovarian cancer cell lines. Ascites samples of cancer patients.	NFV induced cell death in carboplatin-sensitive resistant ovarian cancer cell lines. NFV induced formation of ER-derived vacuoles and induced up- regulation of the hsp70 heat shock family member GRP78. It induced the unfolded protein response, which causes cell cycle arrest and apoptosis. Down-regulation of cell cycle regulatory proteins, especially cyclin D3.
Dewan, 2009 ^43^	Lymphoblastoid B cells *in vitro* and in mice model	Ritonavir induced cell cycle arrest and apoptosis by down-regulation of cyclin D2 and surviving and suppressed transcriptional activation of NF-κB.
Wang, 2010 ^44^	Tumor vascular network	NFV improved vascular network.
Xie, 2011 ^45^	Chemical systems biology	Weak inhibition of multiple kinases is one of the causes of NFV anti-cancer activity, without severe side effects, but still having an impact on the system. Off targets of NFV are possibly: EGFR, IGF-1R, Akt2, Abl, FGFR, CDK2, ARK2, Fak1, PDK1, Ephrin receptors. The concept behind this research is that the whole is greater than the sum of the parts.
Tian, 2011 ^46^	Glioblastoma cells	Authors describe a pathway NFV/ERS/CHOP/up regulation of trail receptor DR5. This pathway clearly interconnects NFV with apoptosis.
Brüning, 2011 ^47^	Cervical cancer line	NFV and Bortezomib (a proteosomal inhibitor) show synergy as apoptosis inducers.
Zeng, 2011 ^48^	Pituitary adenoma cells and xenografted tumors	Growth retardation *in vivo*. Inhibition of PI3K-Akt-mTOR axis. Increased sensitivity to radiation.
Guan, 2012 ^49^	Cell culture	NFV inhibits proteolysis of SREBP-1 by inhibiting site-2 protease (S2P). Inhibition of autophagy with hydroxychloroquine enhanced apoptotic effect of nelfinavir. Accumulation of SREBP-1 produced ER stress. Decreases FAS.
Bono, 2012 ^50^	Human myeloma plasma cells and xenografted SCID mice	Inhibition of S26 proteasome, impaired proliferation and increased apoptosis. Decreased phosphorylation of AKT, STAT3 and ERK ½. ER stress corroborated by PERK and CHOP.
Brüning, 2012 ^51^	HeLa cells and other cancer cells	NFV and other ER stressor upregulate inhibin Beta E which shows anti- proliferative effects.
Barillari, 2012 ^52^	Cervical intraepithelial neoplasia cells	Saquinavir and ritonavir reduce MMP2 and 9, inhibiting cell invasion and growth.
Timeus, 2012 ^53^	Neuroblastoma cells	Saquinavir showed anti-proliferative and anti-invasive activity which was increased by the association with imatinib. Activation of NF-κB was decreased. Saquinavir also exerted a pro-apoptotic activity on NB lines, which was significantly increased by the association with imatinib.
Ismail, 2013 ^54^	Hamster ovary cells	Inhibits proximal insulin receptor signalling which may explain insulin resistance.
Escalante, 2013 ^55^	Myeloma cells in culture and mice xenografts.	NFV is a calpain blocker. This activity enhances bortezomib (a proteosomal inhibitor) citotoxicity *in vivo* and *in vitro*. Drugs that block the HIV1-associated aspartyl protease also show cross reactivity with the cysteine protease calpain.
Bociaga-Jasik, 2013 ^56^	Pre-adipocytes and adipocytes in culture	Saquinavir decreased mitochondrial membrane potencial and intracellular ATP in adipocytes.

## Nelfinavir and the ERS-UPR pathway

NFV inhibits the proteases S1P and S2P that are involved in SREBP-1 maturation and other proteases necessary for protein maturation and folding (yet not fully identified) in the endothelial reticulum ^49^.

Activation of the unfolded protein response (UPR) starts in the ER when abnormal accumulation of protein is detected ^57^. This was investigated thoroughly in yeasts where detection of abnormal protein occurs through Ire1p/Ern1p-mediated signaling from the ER (in mammals there are three sensor proteins IRE1α, PERK and ATF6 ^58^). UPR activation leads to the specific removal of 252 nucleotides intron from a precursor mRNA of the transcription factor HAC-1p, and the resulting mature mRNA HAC-1p is translated to produce active HAC-1p. This transcription factor translocates to the nucleus and promotes the transcription of chaperones like GRP78 that facilitates removal of abnormal proteins from the ER through retrotranslocation and final disposal by the ubiquitin-proteasome pathway ^59^.

HAC1 precursor mRNA is constitutively expressed but not translated until Ire1p/Ern1p sensor removes the necessary nucleotides.

Thus the UPR is an intracellular signaling pathway where the ER “informs” the nucleus on the need to increase the levels of molecular chaperones and folding enzymes in order to maintain the ER homeostasis. Therefore UPR keeps unfolded proteins in the ER until they are correctly folded before they can go to their final destination. NFV seems to produce cellular stress by accumulation of misfolded or abnormal proteins in the ER, overwhelming the normal ER protein folding machinery ^60^. Chaperones bound to unfolded proteins in the ER initiate protein kinase cascades that inhibit translation, reverse translocation, activate ubiquitination enzymes, induce autophagia, and when stress is extreme, induce apoptosis.

## Mechanism of action of Nelfinavir in cancer

**Figure 1.**
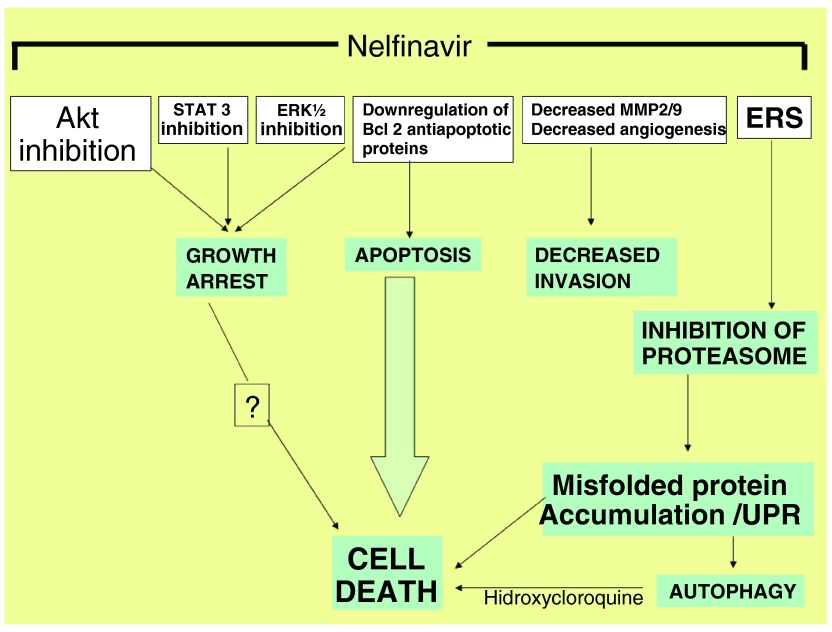
Simplified mechanisms of action of nelfinavir in cancer. ERS: Endoplasmic reticulum stress.

**Figure 2.**
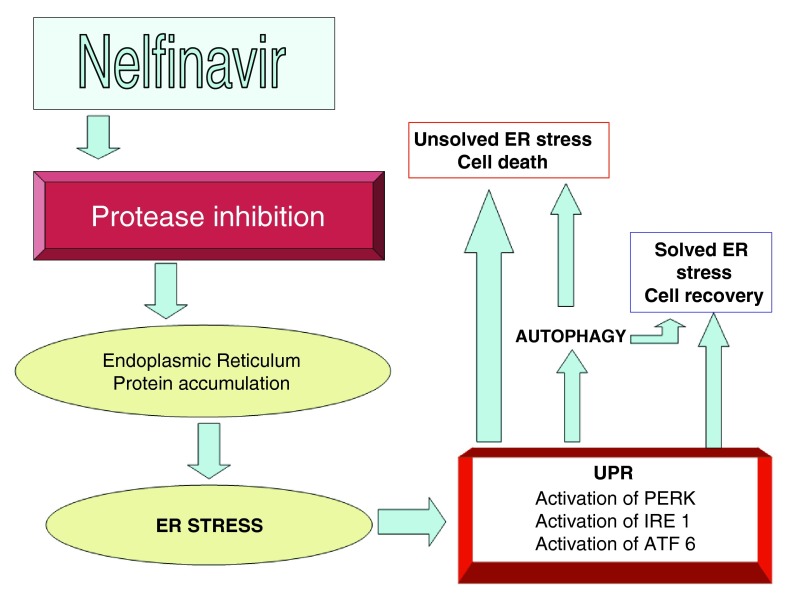
A more detailed view of nelfinavir’s action on ER stress.

**Figure 3.**
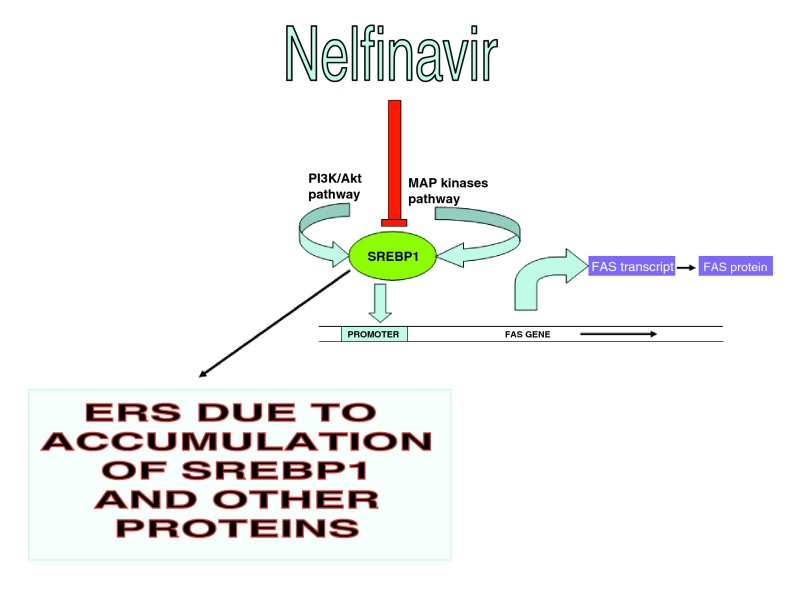
Nelfinavir inhibition of SREBP1 as a cause of endoplasmic reticulum stress (ERS). For this figure the model of nelfinavir in liposarcoma was used.

**Figure 4.**
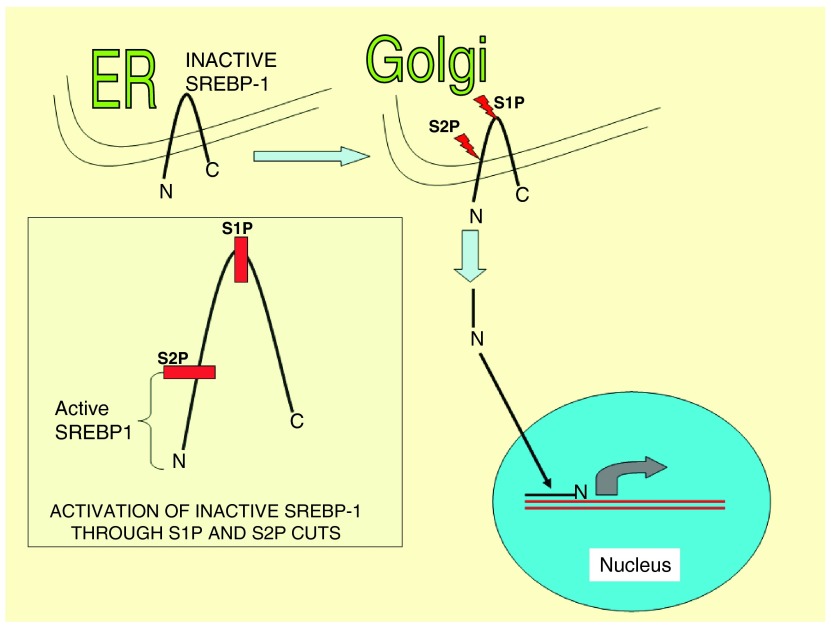
SREBP is synthesized as an ER transmembrane protein and transported to the Golgi upon appropriate stimulus. For activation of SREBP it is necessary that luminal S1P (a protease) cleaves first, followed by intramembrane S2P (another protease) to liberate the transcriptionally active amino-terminal segments of nSREBP. NFV inhibits S1P and S2P, so that transcriptionally active SREBP is not produced. Accumulation of inactive SREBP is one of the UPR initiators.

**Table 2.** Nelfinavir anti-cancer activity in different tumor tissues*.

Tumor	Reference	Level
Hepatocarcinoma	Sun, 2014 ^62^	Cell culture
Diffuse B cell lymphoma	Petrich, 2012 ^63^	Cell culture
Glioblastoma	Kast, 2012 ^64^	Cell culture
Liposarcoma	Pan, 2012 ^65^	Clinical Trial (Phase I)
HER 2 positive, breast cancer cells	Shim, 2012 ^66^	Cell culture
Acute myeloid leukemia	Kraus, 2014 ^67^	Cell culture
Acute myeloid leukemia	Kraus, 2013 ^68^	Cell culture
Cancer stem cells expressing embryonic genes**	Darini, 2013 ^69^	Cell culture
Glioblastoma	Kas, 2013 ^70^	Clinical
Castration resistant prostate cancer	Mathur, 2014 ^71^	Cell culture
Medullary thyroid cancer	Kushchayeva, Jensen, Recupero 2014 ^72^	Cell culture
Medullary thyroid cancer	Kushchayeva, Jensen, Burman, 2014 ^73^	Cell culture
Glioblastoma	Alonso-Basanta, 2014 ^74^	Clinical Trial, Phase I
Rectal cancer	Buijsen J, 2013 ^75^	Clinical Trial, Phase I
Refractary adenoid cystic carcinoma	Hoover, 2014 ^76^	Clinical Trial, Phase II

*Saquinavir-NO has been tested in human melanoma cells with good results ^77^. **It is necessary to underscore the finding that cancer stem cells expressing embryonic genes like Oct4, Sox2 and others, are particularly prone to apoptosis when PIs are used, particularly iopinavir (nelfinavir and saquinavir are also effective in this matter).

**Table 3.** Summary of clinical trials performed with NFV as an anti-cancer drug.

PROTOCOL	CHARACTERISTICS
NCT 0145106	**SOLID TUMORS:** A phase I trial of nelfinavir (Viracept ) in adults with solid tumors
NCT 00436735	**SOLID TUMORS:** Nelfinavir in treating patients with metastatic, refractory, or recurrent solid tumors
NCT 02080416	Non-Hodgkin Lymphoma, Hodgkin Lymphoma, Kaposi Sarcoma, Gastric Cancer, Nasopharyngeal Cancer, EBV, Castleman Disease: nelfinavir for the treatment of gammaherpesvirus-related tumors
NCT 00589056	**NSCLC:** NFV, radiation therapy, cisplatin, and etoposide in treating patients with stage III non- small cell lung cancer that cannot be removed by surgery
NCT 01068327	**Pancreatic cancer:** stereotactic radiation therapy, nelfinavir mesylate, gemcitabine hydrochloride, leucovorin calcium, and fluorouracil in treating patients with locally advanced pancreatic cancer
NCT 01925378	**Cervical intraepithelial neoplasia:** a phase II single-arm intervention trial of nelfinavir in patients with grade 2/3 or 3 cervical intraepithelial neoplasia
NCT 01959672	**Pancreatic cancer:** nelfinavir mesylate in treating patients with locally advanced pancreatic cancer
NCT 01079286	**Renal cell cancer:** study of nelfinavir and temsirolimus in patients with advanced cancers
NCT 00704600	**Rectal cancer:** nelfinavir, a phase I/phase II rectal cancer study
NCT 01065844	**Adenoid cystic cancer :** nelfinavir in recurrent adenoid cystic cancer of the head and neck
NCT 01164709	**Hematologic cancer:** nelfinavir mesylate and bortezomib in treating patients with relapsed or progressive advanced hematologic cancer
NCT 01086332	**Pancreatic cancer:** evaluation of nelfinavir and chemoradiation for pancreatic cancer
NCT 01485731	**Cervical cancer:** safety study of nelfinavir + cisplatin + pelvic radiation therapy to treat cervical cancer
NCT 02024009	**Pancreatic cancer:** systemic therapy and chemoradiation in advanced localised pancreatic cancer - 2
NCT 00915694	**Glioblastoma:** nelfinavir, radiation therapy, and temozolomide in treating patients with glioblastoma multiforme
NCT 01108666	**NSCLC:** proton beam radiation with concurrent chemotherapy and nelfinavir for inoperable stage III non small cell lung cancer (NSCLC)
NCT 00791336	Study to evaluate using nelfinavir with chemoradiation for NSCLC
NCT 02207439	**Larynx carcinoma**: A phase II of nelfinavir, given with definitive, concurrent chemoradiotherapy (CTRT) in patients with locally-advanced, human papilloma virus (HPV) negative, squamous cell carcinoma larynx
NCT 01020292	**Glioma:** nelfinavir and concurrent radiation and temozolomide in patients with WHO grade IV glioma
NCT 01555281	**Myeloma**: nelfinavir and lenalidomide/dexamethasone in patients with progressive multiple myeloma that have failed lenalidomide-containing therapy
NCT 01728779	**Metastatic lesions of the lung, liver or bone**: stereotactic body radiation with nelfinavir for oligometastases.
NCT 00233948	**Liposarcoma:** nelfinavir mesylate in treating patients with recurrent, metastatic, or unresectable liposarcoma
NCT 02188537	**Myeloma:** nelfinavir as bortezomib-sensitizing drug in patients with proteasome inhibitor- nonresponsive myeloma
EudraCT Number: 2008-006302-42 University of Oxford	**Pancreatic cancer:** a phase II study in patients with locally advanced pancreatic carcinoma: ARC-II – Akt-inhibition by nelfinavir plus chemoradiation with gemcitabine and cisplatin

**Figure 5.**
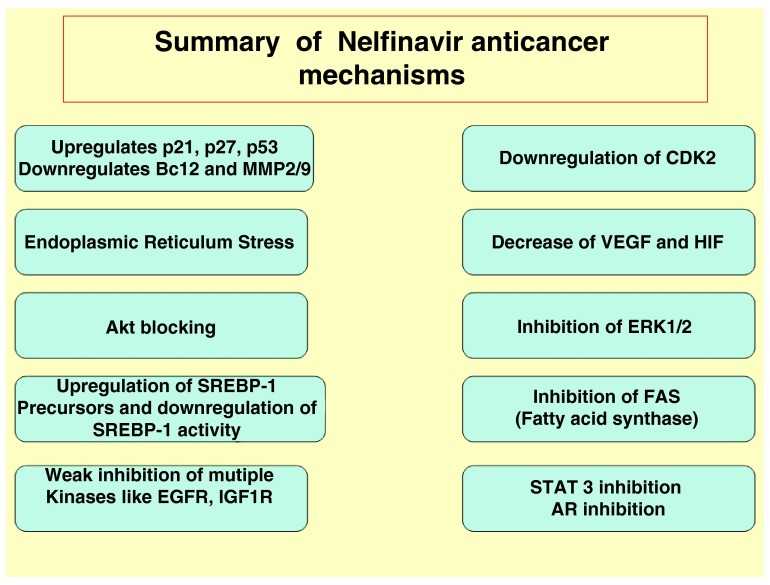
Summary of NFV anti-cancer mechanisms.

## Interactions of NFV and PIs with other drugs

Indinavir and NFV increase anti-malarial action of artemisinin *in vitro* on *Plasmodium falciparum* ^78^, but artemisinin has also an off target anti-cancer activity. Thus it is reasonable to raise the question: may the association of artesiminin with NFV increase anti-cancer activity?

Another research team ^70^ has included both, NFV and artesiminin, in a multidrug repurposed protocol (CUSP 9) for the treatment of relapsed glioblastoma.

Celecoxib is an ER stressor that may enhance NFV anti-tumor activity ^79^.

Chloroquine and hydroxicloquine are autophagy inhibitors and may work synergistically with NFV, down-regulating autophagy and increasing apoptosis ^70, 80^.

Nelfinavir may produce overproduction of mcl1 through upregulation of Erk ½, which would reduce apoptosis. The problem can be solved adding sorafenib ^81, 82^.

In breast cancer cells, tamoxifen enhances anti-cancer activity of NFV ^83^. This synergism was independent of the estrogen receptor status so that the authors consider that the association of NFV and tamoxifen may be advantageous even in patients with no hormone responsive tumors.

Saquinavir has an interesting off target effect: it decreases intracellular ATP in adipocytes ^56^. If this effect is similar in tumor cells, an association with metformin and 2-deoxyglucose may produce anti-cancer activity ^84– 86^. There is growing interest on metabolic perturbators in cancer therapy and saquinavir may play a role in this field.

## Clinical trials

A phase I dose escalation trial performed in 2014 ^87^ established a MTD (maximum tolerated dose) of 3125 mg twice daily and described that 45% of patients with solid tumors treated with this dose decreased AKT activity and increased ERS indicators. This indicated a possible benefit in neuroendocrine tumor patients and also established that dose limiting toxicity consists in neutropenia.

The dose (3125 mg bid) is more than twice the dose used in HIV treatment. But lower doses, in the range of those used in HIV treatment have been tested, combining nelfinavir with chemoradiotherapy in pancreatic cancer with evidence of efficacy ^88^. No control group was used in this research, so comparison was established with known data from previous publications, mainly the favourable possibility of tumor resection after treatment.

Even lower doses (625 mg and 1250 mg bid) were tested in a phase I trial of NSCLC in stages IIIA/IIIB combined with chemoradiotherapy ^89^. In nine out of 12 patients a PET scan was available post-treatment with 100% overall response (56% complete response and 44% partial response). Unfortunately, in this trial there was no control group; 50% of the patients (six out of 12) lived for more than 22 months after treatment; 25% (three out of 12) lived without disease for more than 32 months. The results may be considered favourable, even without a control group.

Buijsen *et al.* ^75^ recommend a dose of 750 mg NFV bid for a phase II trial of this drug in combination with chemoradiotherapy in locally advanced rectal cancer.

Ongoing phase II trials are mainly in myeloma (associated with bortezomib or lenalomide), glioblastoma patients (associated with chemoradiation), pancreas (associated with gemcitabine and radiation) and lung. (See http://www.clinicaltrials.gov).

The first results of clinical trials did not show a meaningful improvement in the outcome of patients with refractory adenoid cystic carcinoma which is a malignant salivary gland tumor which usually has a poor prognosis. In this case NFV was used as monotherapy ^76^.

## Why nelfinavir has a role to play in cancer therapy?

Akt activation is an important step in cancer phenotype and is a key player in acquisition and maintenance of cancer hallmarks. Akt is a nodal regulator of cellular survival pathways ^90^. There are no drugs at the present time that can inhibit this protein with a good safety profile. Wortmannin, perifosine and other chemicals designed for PI3K/Akt inhibition were too toxic for clinical use or have shown disappointing results, so they did not enter the medical practice. Insulin stimulation of Akt phosphorylation was reduced by 55% at achievable doses ^20^. At the same time there is clear evidence that it favours apoptosis and growth inhibition at clinically tolerable and achievable doses.

This anti-Akt activity of NFV can be reinforced by concomitant mTOR inhibition which results in synergistic cytotoxicity ^63^. This may be due to the fact that mTOR inhibition without Akt inhibition eliminates a negative biofeedback loop on Akt, producing increased phoshorilation of Akt ^91^. According to Sarbassov ^92^ this negative feedback is born in the mTORC2 complex.

**Figure 6.**
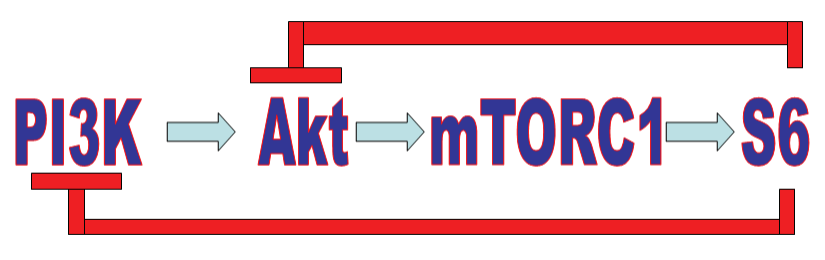
Negative feedback loop.

According to Carracedo ^93^, this negative feedback loop goes as far as PI3K ( Figure 6).

mTOR inhibitors have become a new and important tool against cancer, for example in renal cell carcinoma. But the negative biofeedback loop on Akt must be solved to achieve really good results. NFV could be the clue.

**Figure 7.**
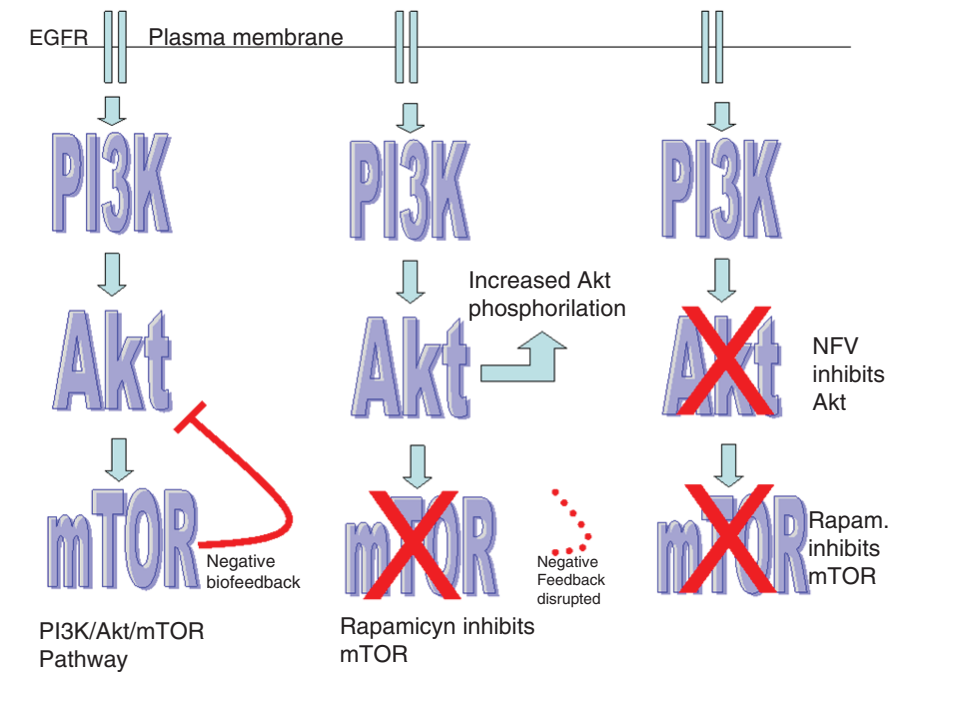
Mechanism of synergy between NFV and mTOR inhibitors. At the left is depicted the pathway under normal or pathological circumstances; in the middle drawing mTOR inhibition is counterbalanced by Akt activation due to loss of the negative biofeedback circuit; on the right, inhibition of both, mTOR and Akt may result in increased anti-cancer results.

But the most important anti-tumor activity of NVR is not limited to Akt inhibition but ER stress and UPR which may be one of the pathways leading to apoptosis ^94^.

Additional features of NFV and other protease inhibitors are

1) the ability to sensitize cancer cells to chemoradiotherapy,
2) anti-angiogenesis by decreasing VEGF/HIF expression,
3) decreased expression of FAS (fatty acid synthase),
4) the combination of radiation and PIs is well tolerated ^39, 94^,
5) NFV cancer cell killing ability can easily be enhanced with other ER stressors like celecoxib ^79^. Cho *et al.* found enhanced killing of chemoresistant breast cancer cells after celecoxib treatment that aggravated ER stress; perillyl alcohol is another stress aggravator that has been used with that purpose ^96^,
6) In head and neck cancer related to HPV, NFV produced down-regulation of Akt and radiosensitization ^97^,
7) NFV not only down-regulates Akt but also MAPK (in adenoid cystic cancer) ^98^, and retards oral cell proliferation including normal keratinocytes and squamous cell cancer ^99^,
8) There are evidences, at least in pancreatic cancer, that NFV dependent down-regulation of Akt is independent of the mutational status of K-ras ^100^,
9) There is clear evidence (in glioblastoma) of the relation between NFV and apoptosis through the following pathway ^46^:

NFV------ER stress--------CHOP------up regulation of TRAIL receptor DR5

10) Down-regulation of MMP-9 (reduced expression and secretion of MMP-9 by human preadipocytes) ^64, 101^,
11) Increased apoptosis by NFV when associated with anti-autophagy drugs like chloroquine or hydroxychloroquines, particularly in triple negative breast cancer cells ^102^.

## Possible controversies

The SREBP pathway for regulation of fat metabolism is initiated through proteolytic cleavage of precursor forms of the SREBPs (125 Kd protein) in ER membranes. When cells are in need of sterol, the precursor SREBPs are hydrolyzed by a 2-step mechanism involving membrane-bound serine protease S1P and a metalloprotease S2P. The N-terminal fragment of SREBP (nSREBP) is a 68 Kd protein that translocates to the nucleus where it works as a promoter-enhancer, binding to sterol regulatory elements located in DNA and activates gene transcription ( Figure 4). The nuclear SREBP can be rapidly degraded by a proteasome-mediated mechanism. This provides regulation of gene transcriptional activities ^103^.

Transgenic mice over-expressing the constitutively active nuclear forms of the SREBPs (nSREBPs) revealed that overexpression of SREBP-1 or SREBP-2 leads to activation of genes involved in the cholesterol and fatty acid biosynthesis cascades. These transgenic mice displayed the classical features of generalized lipodystrophy, similar to those found in patients under PI therapy ^104^.

Riddle *et al.* in 2001 ^105^ found that PI therapy (they used ritonavir) induced the accumulation of activated SREBP-1 and SREBP-2 in the nucleus of liver and adipose tissues. As a consequence, fatty acid and cholesterol biosynthesis were increased in these tissues. The authors consider that lipodystrophy, hyperlipidemia, and insulin resistance, are the consequence of activated SREBP-1 and SREBP-2 accumulation in the nucleus of liver and adipose tissues. The possible mechanism for these events, according to their criteria is PI suppression of activated SREBP degradation in the nucleus. In summary, Riddles’s study showed that ritonavir induced lipid metabolism abnormalities through stabilization of activated SREBP-1 and SREBP-2 in the nucleus of liver and adipose tissues.

These findings are in contrast with those of Guan ^49, 106^ where NFV inhibited the nuclear translocation of the sterol regulatory element binding protein-1 (SREBP-1) in castration resistant prostate cancer and liposarcoma through inhibition of S1P. This led to accumulation of unprocessed SREBP-1.

Riddle *et al.* described accumulation of processed SREBP-1 in the liver and adipose tissue while Guan found accumulation of unprocessed SREBP1 in ER and Golgi with no translocation to nucleus in liposarcoma and castration resistant prostate cancer tissue.

The controversy may be explained in the following way:

1) There are three different isoforms of SREBP: SREBP-1a, SREBP-1c and SREBP-2.
2) SREBP-1a and -1c have different expression profiles: SREBP-1a is highly expressed in proliferating cells, such as cancer cells, while SREBP-1c is the predominant form in normal cells, particularly hepatocytes ^104^.
3) The target genes for the three SREBP isoforms are different.
4) Riddle *et al.* found increased SREBP-1 and two in the nucleus of liver and adipose tissues; these SREBPs are the active form (they make no difference between SREBP-1a and SREBP-1c).
5) Guan *et al.* found increased SREBP in Golgi in the inactive form (precursor) of tumor tissues treated with NFV.
6) It is possible that tumor tissues that overexpress SREBP-1a behave in a different way than liver and adipose tissue that overexpress SREBP-1c.
7) Riddle *et al.* tested ritonavir and Guam *et al.* tested NFV, so the pharmacological effects between these PIs may differ.

A second controversy that stems from the one described above is on the effect of NFV on FAS:

1) According to Guan *et al.* ^106^, NFV decreases expression of FAS in liposarcoma cells and castrate resistant prostate cancer as was depicted in Figure 3.
2) According to Lenhard *et al.* 2000 ^107^, NFV increases expression of FAS in HepG2 cells (which show many of the normal biochemical functions of non tumor liver parenchymal cells).

May this difference be due to tissue-specific effects of NFV? Does NFV have different effects in tumor tissues and normal tissues?

To definitely solve these controversies, it is necessary to proceed with further experimental research, but the findings described above necessarily raise the doubt that mechanisms that work in tumor cells might be slightly different from those working in hepatocytes and adipocytes.

## Possible negative aspects of PIs in cancer

Despite the anti-cancer activity of NFV and PIs, these drugs do not reduce the risk of developing cancer in HIV population ^108^ and also exert certain depression of immunological functions, interfering with the differentiation program of monocytes into dendritic cells ^109^.

PIs increase the expression of P-glycoprotein (ABCB1) in Kaposi’s sarcoma cell lines increasing the multidrug resistance phenotype ^110^. At the same time ABCB1 expression depends on Akt activation ^111^ and NFV inhibits partially Akt. The final result of the two antagonistic aspects requires further research.

There are well known undesirable side effects with HIV PIs, like hyperlipidemia, insulin resistance and lypodystrophy (peripheral fat wasting and excessive central fat deposition). One of the main responsible mechanisms of these side effects is the suppression of the breakdown of SREBP in the liver and adipose tissues resulting in increased fatty acid and cholesterol biosynthesis. SREBP accumulation in adipose tissue causes lipodystrophy.

PIs suppress proteasome-mediated breakdown of nascent apolipoprotein (apo) B, resulting in the overproduction of triglyceride. Finally, PIs also suppress the inhibition of the glucose transporter GLUT-4 activity in adipose tissue and muscle. This contributes directly to insulin resistance and diabetes ^112^.

Hepatomegaly and hepatic steatosis are direct consequences of the metabolic alterations explained above ^105^.

## New PIs with anti-cancer activity

In 2010 You *et al.* ^113^ synthesized a new indinavir analogue with remarkable anti-cancer activity, similar to NFV: CH05-10. This drug achieved similar cytotoxity to NFV but at lower concentrations, against leukaemia, melanoma, ovarian and prostate cancer cell lines.

In 2009 Saquinavir-NO was introduced ^114^; it showed interesting anti-cancer properties in melanoma xenografts with significantly lower toxicity than saquinavir.

## Conclusions

The most relevant mechanisms of PIs anti-cancer activity are Akt inhibition and ER stress.

Following our exhaustive analysis of the current medical literature we conclude that NFV anti-cancer activity is mainly dependent on ER stress-UPR.

Akt inhibition plays also a very important role but is not the unique or main source of anti-cancer effects.

The evidences that support these conclusions are:

1) Even at very high doses of NFV (3,125 mg bid), Akt achieved a level of inhibition around 55% in cell culture ^20^.
2) NFV is at the same time a strong ER stressor and an Akt inhibitor.
3) Anti-cancer activity can be achieved at much lower doses than those necessary for Akt inhibition.
4) Increasing ER stress by adding Celecoxib to NFV enhances cytotoxicity.
5) Autophagy, which is one of the mechanisms cells use to survive increasing ER stress ^115^, is inhibited by adding chloroquine or hydroxychloroquine to NFV. In this case, apoptosis is significantly enhanced ^80, 102^.
6) The PIs with anti-cancer activity like NFV, ritonavir ^116^, saquinavir ^117^, and the experimental drug CH05-10 ^113^ are strong ER stressors. Amprenavir is a PI that induces no ER stress and its anti-cancer activity is significantly weaker than that of NFV, although it has Akt inhibiting effects.
7) Ritonavir, which is ER stressor, shows anti-cancer activity although it does not down-regulate Akt at concentrarions usually found in HIV patients ^23^.
8) Inhibition of proteasome with bortezomib has a synergistic effect with NFV apoptotic activity ^118^
9) PIs can be classified regarding anti-cancer activity at clinically achievable concentration in patients in
  A) Akt inhibitors only: e.g. amprenavir
  B) ER stressors only: e.g. ritonavir
  C) Akt inhibitors and ER stressors: examples NFV and experimental PI CH05-10. Of course this is the group that shows stronger anti-cancer activity.

There is enough evidence of NFV anti-cancer effects and there is adequate knowledge of how this activity works, so that NFV deserves well designed phase II clinical trials, as adjunct cancer therapy.

Associations with proteasomal inhibitors, celecoxib and other cell stressor should also be investigated in the clinical setting due to possible synergy. Tamoxifen with NFV may show interesting results in breast cancer.

Although a large amount of publications, including reviews, have been written on NFV and other PIs in cancer, none has been dedicated to a thorough examination and analysis of the mode of action of these pharmaceuticals as off target drugs (with the exception of the review by Gantt *et al.* ^2^). It is hoped that this review will encourage an increment adequately powered and well-designed clinical trials in the various cancer types, beyond the phase I trials that have been recently performed, and specifically trials where these compounds may be tested in association with other known anti-cancer pharmaceuticals like NFV associated to bortezomib and hydroxychloroquine in myeloma, or mTOR inhibitors with NFV in HNSCC and many other possible combinations where the dual feature of NFV, ER stressor and Akt inhibitor, are required.

In myeloma NFV increases proteasome inhibition by bortezomib and may overcome resistance to proteasomal inhibitors. This is an action exclusive of NFV and not shared with other PIs ^67^, with the additional advantage that NFV shows the highest cytotoxic activity against primary myeloma cells.

If apoptosis is described as a cascade, then apoptosis stimulator drugs like NFV should be viewed as enhancers of this cascade. An initiator of the cascade is still necessary, for example chemoradiotherapy. After this initial step, apoptosis stimulator drugs increase the amount of cells entering this pathway. This might be one of possible reasons why nelfinavir alone has shown poor results in a clinical trial used as monotherapy.

This does not mean that NFV cannot act as an initiator, but the evidences show that it is prone to be an enhancer of apoptosis rather than an initiator.

## Future directions

All the evidences presented in this review reinforce the concept that NFV is a useful drug in cancer treatment. It should be considered in association with chemoradiotherapy in the design of new protocols for diseases like multiple myeloma (in association with bortezomib and hydroxychloroquine) and prostate, pancreas and lung cancer where clinical trials are ongoing. New PIs are being developed with better anti-cancer profile like CH05-10 and saquinavir-NO ^77^ and further development of new PIs with stronger anti-cancer activity, will probably go on in the future.
