# Supplementary material for: Nelfinavir and other protease inhibitors in cancer: mechanisms involved in anticancer activity
Source: F1000Res. 2015 Mar 5;4:9. Originally published 2015 Jan 12. [Version 2] doi: 10.12688/f1000research.5827.2 (PMC4457118; doi:10.12688/f1000research.5827.2)
Supplement: Peer review history

# Peer review: Referee response for version 2

**DOI:** 10.5256/f1000research.6594.r8788

**Published:** 2015-06-01

**Authors:** Ansgar Brüning

**Affiliations:** 1 Molecular Biology Laboratory, University Hospital Munich, Munich, Germany

This is a highly informative and comprehensive review on our current knowledge about the anti-cancer mechanism of nelfinavir. It also deals with the pros and cons of a possible repurposing of nelfinavir (and other HIV PIs) for cancer treatment.

The following amendments and corrections are suggested:

Proteasome: In the abstract, the proteasome is listed among the factors to be “down-regulated” by nelfinavir. Is the proteasome “downregulated” or “inhibited” in its activity?  This might be a slight semantic inaccuracy but is important because it reflects different molecular mechanisms. The same applies to Akt, which, according to the author, is mentioned to be “inhibited” in some phrases and to be “downregulated” (as a protein?) by nelfinavir in other phrases.

Independent of this, there is also a controversy in the literature about the ability of nelfinavir to inhibit proteasomal activity. Some authors observed inhibition of proteasomal enzyme activity by HIV PIs, whereas proteasomal target genes such as HIFalpha and cyclins have unexpectedly not been described to accumulate in the presence of PIs. Instead, as mentioned in this review, several of these proteasomal target proteins were found to be downregulated by nelfinavir treatment.

Using yeast terminology (HAC1) to introduce human ER stress mechanisms might confuse the reader.

Fig.1: How does ER stress lead to proteasome inhibition?

I have problems with the author’s proposed classification of PIs into ER stress inducers and Akt inhibitors (Abstract and Conclusion). Ritonavir is listed as an ER stressor only (conclusions “9”), but has been identified by several researchers to cause both ER stress and Akt inhibition. Furthermore, according to our experience, the ability of PIs to reduce Akt activity appears to be dependent on the cell line(s) used. Our group also observed enhanced Akt phosphorylation by nelfinavir in cell lines with low endogenous Akt activity, and Akt activation is also a known response to ER stress.

A combination of nelfinavir with mTOR inhibitors is suggested by the author. There are some independent recent reports indicating that nelfinavir also acts as an mTOR inhibitor.

The ability of nelfinavir to induce oxidative stress should be mentioned, because this may represent one of the reasons why nelfinavir may exert ER stress or other anti-cancer effects. (Fig. 2, for example, suggests undefined “protease inhibition” as the only mechanism of nelfinavir-induced ER stress).

Some typos in the text and in the figures should be corrected (hidroxychloroquine; phosphorilation; endothelial (=endoplasmic?) reticulum; Bc12 (=bcl2? Fig. 5)).

I have read this submission. I believe that I have an appropriate level of expertise to confirm that it is of an acceptable scientific standard.

# Response

**Published:** 2015-06-03

**Authors:** Tomas Koltai

**Affiliations:** Centro de Diagnostico y Tratamiento OSPIA Zona Norte, Argentina

Thank you for your deep and knowledgeable revision of this manuscript. I agree with you about the inaccuracies mentioned and there will be corrected in a third version of the article.

Of course, down-regulation and inhibition in the molecular world are two different things, so it is wise to make the modifications you suggested: proteasome is inhibited and not down-regulated by NFV. Akt, being a protein is down-regulated and not inhibited. These will be modified in version 3.

Yeast terminology for proteins playing a role in proteosomal stress may be confusing, so it will be changed for proteins found in mammalian proteosomal stress.

The classification of PIs according their main anticancer activity is based on clinically achievable concentration of the drug. It is quite possible (I found no experimental evidence so far) that at very high concentrations, PIs are both ER stressors and Akt down-regulators. But at doses used in the clinical setting the classification I propose may represent actual differences between the different PIs.

Oxidative stress will be mentioned in version 3. I agree it is an important cause of ER stress.

Amendments will be made to figure 5 along with the remarks you kindly made.

The association of NFV and mTOR inhibitors showed interesting results in naturally occurring squamous cell tumors in dogs in our experience (unpublished data). This probably means some sort of synergy between the two. This synergy is mentioned by many authors. NFV acting on mTOR will be particularly highlighted.

Thank you for your interesting revision. All issues you mentioned will be considered in version 3 of this article.

# Peer review: Referee response for version 1

**DOI:** 10.5256/f1000research.6229.r7500

**Published:** 2015-02-10

**Authors:** Adali Pecci

**Affiliations:** 1 Catedra de quimica biologica, Facultad de Ciencias Exactas y Naturales, Universidad de Buenos Aires, Buenos Aires, Argentina

The article by Koltai reviews the extensive literature concerning Nelfinavir as an anti-cancer drug. Overall the study makes a good update of the evidences of nefilnavir anti-cancer activity and its role in controlling ERS-UPR pathway. It would be interesting to include in the discussion information about  the different trials that are currently carry on with Nelfinavir in different tumors.

I have read this submission. I believe that I have an appropriate level of expertise to confirm that it is of an acceptable scientific standard.
